# Featured Organism: *Schizosaccharomyces pombe*, The Fission Yeast

**DOI:** 10.1002/cfg.92

**Published:** 2002-04

**Authors:** Jo Wixon

**Affiliations:** Bioinformatics Division HGMP-RC Hinxton, Cambridge CB10 1SB UK

## Abstract

*Schizosaccharomyces pombe*, the fission yeast, has long been a crucial model for the study of the eukaryote cell cycle. We take a look at this important yeast, whose genome has recently been completed, featuring comments from Valerie Wood, Jürg Bähler, Ramsay McFarlane, Susan Forsburg, Iain Hagan and Paul Nurse on the implications of having the complete sequence and future prospects for *pombe* genomics.


**Comparative and Functional Genomics**
*Comp Funct Genom* 2002; **3**: 194–204.
Published online 5 March 2002 in Wiley InterScience (www.interscience.wiley.com). **DOI:** 10.1002/cfg.92

**Feature**

### Featured Organism: *Schizosaccharomyces pombe*, the fission yeast

Jo Wixon*, Managing Editor
*Bioinformatics Division, HGMP-RC, Hinxton, Cambridge, CB10 1SB, UK*

*Correspondence to: J. Wixon, Bioinformatics Division, HGMP-RC, Hinxton, Cambridge, CB10 1SB, UK.*

### Abstract

***Schizosaccharomyces pombe*, the fission yeast, has long been a crucial model for the study of the eukaryote cell cycle. We take a look at this important yeast, whose genome has recently been completed, featuring comments from Valerie Wood, Jürg Bähler, Ramsay McFarlane, Susan Forsburg, Iain Hagan and Paul Nurse on the implications of having the complete sequence and future prospects for *pombe* genomics.** **Copyright © 2002 John Wiley & Sons, Ltd.**

### Summary

- **Fission yeast; reproduces by medial fission rather than budding**
- **13.8Mb genome, as three chromosomes**
- **Genome sequence completed, ~5000 genes**
- **~10% of the predicted genes are orphans**
- **No signs of whole genome duplication like that seen in *S. cerevisiae***
- **Evolutionarily very distant to *S. cerevisiae***

### Background

First described in 1893 by P.Lindner, *Schizosaccharomyces pombe* was named from '*pombe*', the Swahili word for beer, since it was originally isolated in millet beer from eastern Africa. It was called fission yeast, since it was observed to reproduce by fission alone, with no budding like that seen in the brewer's yeast *Saccharomyces cerevisiae*. *S. pombe* cells are usually cylindrical, with rounded ends (Figure 1), but upon starvation the cells shorten and could easily be mistaken for *Saccharomyces* cells. Under certain conditions, pseudo-hyphal growth occurs, in which long tubes form, with numerous transverse walls, or septa. Septa are also formed during normal mitotic growth, prior to fission, becoming the site of a constriction and finally of the separation into two cells. When cells of the two opposite mating types (+ and −) meet, under certain conditions, namely nitrogen starvation, mating occurs and a diploid zygote is formed. Zygotes usually proceed immediately into meiotic division, producing four haploid spores surrounded by an ascus wall.

The bulk of research work done in *S. pombe* is aimed at understanding the cell cycle, for many stages of which *S. pombe* is widely accepted as a better model than *Saccharomyces cerevisiae* for higher eukaryotes. Work done in *S. pombe* has greatly improved our understanding of the eukaryotic cell cycle (Nurse, 2000) and its regulation (Moser and Russell, 2000), and contributed to our knowledge of many related areas, such as microtubule formation (Hagan and Peterson, 2000), meiotic differentiation (Yamamoto *et al.*, 1997), cellular morphogenesis (Brunner and Nurse, 2000) and polarity (Bähler and Peter, 2000), stress response mechanisms (Toone and Jones, 1998), and the response to DNA damage (Zhou and Elledge, 2000).

### Tools for study

The main benefit of a single cell eukaryote is amenability to genetic analysis. This is particularly applicable to *S. pombe* because of its ease of growth, haploid lifestyle and the fact that it is

Copyright © 2002 John Wiley & Sons, Ltd.

Comparative and Functional Genomics (CY74990_8.3d) 12/4/02 11:23:14 *(beta)*
rev: 6.06e/W (Aug 31 2000) The Charlesworth Group, Huddersfield, 01484 517077 CFG92

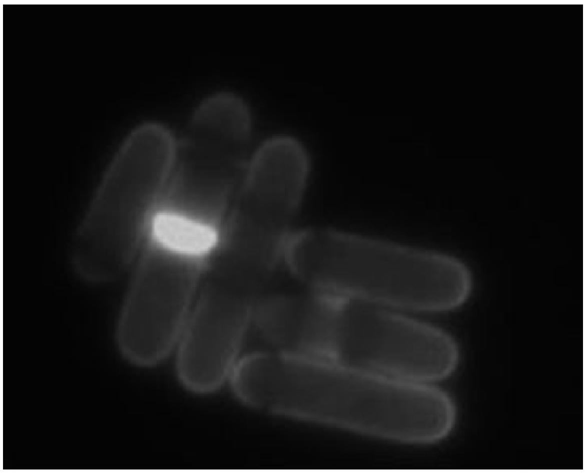

**Figure 1.** Wild-type *Schizosaccharomyces pombe* cells after growth in yeast extract medium at 36°C for 3 hours and staining with calcofluor to highlight cell walls, and septa (in dividing cells). This image was captured by Fiona MacIver and is reproduced by kind permission of Iain Hagan.

amenable to molecular analysis (see below). Most mutants in *S. pombe* are recessive and are either defective in non-essential genes (for example DNA repair genes which, when mutated, leave cells sensitive to treatments with particular genotoxic agents) or in essential genes. When a mutation is in an essential gene it must be a conditional mutation, i.e. only affecting protein function under one condition and not another. Most commonly this condition is temperature, where the mutant protein is non-functional at 36°C (and thus the cell does not grow at 36°C), but functional at 27°C (thus the cell can be propagated at 27°C). Analysis of such temperature sensitive conditional mutants is important because it allows the consequences of sudden loss of a gene function to be examined. Once a mutant in a gene is identified on the basis of phenotype, it is possible to clone complementing genes by transforming yeast with a genomic library or cDNA library constructed in an *E. coli*/yeast shuttle vector and selecting for complementation of mutant phenotype.

Shuttle vector plasmids used for working with *S. pombe* contain origins of replication and selectable markers for use in bacterial systems in addition to a yeast selectable marker (commonly a gene encoding a metabolic enzyme) and an autonomously replicating sequence, which provides a high frequency of transformation. The fission yeast centromere is too big to be easily cloned, hence there are no single copy centromere plasmids; this is a problem for expression studies with genes that are toxic at high expression levels. There are various promoters and fusion or tagging sequences available. The most commonly used promoters in fission yeast are *adh1+* (for constitutive high expression), *fbp1+* (for carbon source responsive expression), a tetracycline-repressible system, and the *nmt1+* (no message in thiamine) promoter, which offers three different levels of expression. In addition, systems have recently been developed for expression of foreign genes in *S. pombe* (Giga-Hama *et al.*, 1999).

LacZ constructs can be used to detect expression of *S. pombe* genes, in conjunction with X-gal plates or $\beta$-galactosidase assays for cells in culture. Gene fusion with the green fluorescent protein (GFP) gene can be used to determine the subcellular localization of *S. pombe* proteins (Atkins and Izant, 1995), for a review, see Sawin (1999). Coupled with advanced fluorescence microscopy techniques, GFP fusions (and other fluorescent marker fusions) can now be used (Haraguchi *et al.*, 1999) to follow chromosome behaviour (Pidoux *et al.*, 2000), or protein localisations and interactions (Bezanilla *et al.*, 2000) in great detail, in living cells.

*S. pombe* cells coordinate their growth with the cell cycle. Exponentially growing wild-type cells are born at a similar cell size and double in mass before entering the next round of cell division. This is due to a size control that couples growth and the cell cycle. Since *S. pombe* cells grow by elongation at the tips, cell length is a measure of the stage of the cell cycle that the cell has reached. Size selection of cells by elutriation (which requires a sophisticated centrifuge and yields enough cells for biochemical analysis) or lactose gradient centrifugation (which is simpler but provides far fewer cells) will yield cells at the same stage in the cell cycle. Another way to achieve a synchronous culture, which is crucial for the study of the cell cycle, is to use temperature sensitive mutants of certain cell cycle genes; once such cells are shifted to the permissive temperature, these cells will all accumulate at the same point in the cell cycle. It is also possible to arrest cells using exposure to certain drugs or pheromone, or nitrogen starvation (see Fission Yeast Handbook, Web-Based Resources).

Traditionally, panels of temperature sensitive (ts) lethal *S. pombe* mutants have been generated by chemical mutagenesis with ethyl methanesulfonate (EMS) or nitrosoguanidine (NG). Once mutants

Copyright © 2002 John Wiley & Sons, Ltd.

Comparative and Functional Genomics (CY74990_8.3d) 12/4/02 11:25:30 *(beta)*
rev: 6.06e/W (Aug 31 2000) The Charlesworth Group, Huddersfield, 01484 517077
CFG92

showing the desired, or novel, phenotypes are identified, the genes carrying the mutation can be identified by complementation analysis. This is the transformation of the mutant strain with a library of *S. pombe* genomic fragments and screening for rescue of the phenotype. The plasmid causing the rescue of the phenotype is then recovered from the strain and the gene complementing the phenotype can be identified. Homologous recombination with PCR-derived constructs can be used for directed insertion of sequences into the *S. pombe* genome; this can be used to delete a chosen gene, or to introduce a marker or fusion construct (Bähler *et al*., 1998). This technique can be used in modified form, with longer flanking homology in the constructs, for deletion of problematic loci (Krawchuk and Wahls, 1999). Suppression of gene expression by antisense RNA is also being explored as a way to knock-out gene function in *S. pombe* (Arndt *et al*., 1995), and *S. pombe* has been suggested as a model for determining which genes will be amenable to this technique in mammalian cells (Clarke *et al*., 2000). Identification of amino acids essential to the function of *S. pombe* proteins can be done by PCR-based random mutagenesis, or by site-directed mutagenesis of chosen codons in the gene of interest.

Systematic analyses of gene expression in *S. pombe* are thin on the ground at the moment, one example studied *S. pombe* gene expression patterns in a ras1 mutant under various conditions (Danjoh and Fujiyama, 1999). A group at the Sanger Institute is currently setting up a microarray for *S. pombe* (see Web-based Resources and Future Aims) using PCR products for each ORF, spotted onto glass slides.

At the '*Pombe* 2000' workshop, held at the ICRF in London, on 1st October 2000, there was a discussion of planned *S. pombe* genomics projects (see The Forsburg lab page, Web-based Resources). This highlighted the existing databases at the Sanger Institute and Proteome Inc. (see Web-based Resources) and discussed planned wet lab projects. In addition to the ongoing project at the Sanger Institute, Tim Humphrey, Yasushi Hiroaka and Karl Ekwall were all considering setting up *S. pombe* microarray facilities. Unfortunately, the *Pombe* Interest Group Genomics Initiative was unable to secure funding for genomic and proteomic analyses from the BBSRC. The attendees felt there was a need to secure the involvement of more people with expertise in the area of proteomics in these *S. pombe* projects. Ramsay McFarlane reported that he and Henk Braig plan to set up a Difference Gel Electrophoresis (DIGE) system at Bangor University (see Future Aims). Yasushi Hiroaka's lab has a GFP-fusion library (see Cell Magic World, Web-based Resources) and the Yoshida group is making an ordered library of full-length ORF clones. Mitsuhiro Yanagida, Bea Grallert and Tony Carr all have ts mutant libraries and discussed the possibility of keeping them together in one place. Recently, John Armstrong and Felicity Watts (Sussex University) have secured Wellcome funding for an *S. pombe* genetic resource center that should include and expand these libraries and facilitate multiple groups to address these and other similar resources. Anabelle Decottignies and Paul Nurse, and Yasushi Hiroaka are performing deletion projects for functional analysis of specific subgroups of *S. pombe* genes.

### Current Status of Genome Knowledge

The haploid *S. pombe* genome is 13.8 Mb in size and is organised into three chromosomes, I (~5.7 Mb), II (~4.6 Mb) and III (3.5 Mb). The systematic sequencing of *S. pombe* was commenced in 1995 at the Sanger Institute, with funding from the Wellcome Trust. This continued until 1996, by which time, two-thirds of chromosome I had been completed. Sequencing was resumed at the end of 1996, by a consortium of 13 European laboratories, headed by the Sanger Institute, with funding from the European Commission. This has resulted in 12.3 Mb of high quality sequence of the complete genome, leaving only the rDNA and a part of each centromeric sequence as gaps (the sequence goes very close to the telomeres, but does not include the telomeric repeats) (Wood *et al*., 2002). Refinement of the existing clone maps was a major part of the sequencing project and this has produced an accurate and reliable minimum clone tile path, which is now one of the key biological resources for the *S. pombe* research community.

The genome is predicted to contain 4940 genes, including the 'very hypotheticals'; these are the ~110 ORFs which score poorly on several criteria, and are thought least likely to be actual genes. These and the ~610 orphans (those predicted genes with no functional data or sequence homology) make up the *S. pombe* specific portion of the predicted genes. There are 11 intact transposable

Copyright © 2002 John Wiley & Sons, Ltd.

Comparative and Functional Genomics (CY74990_8.3d) 12/4/02 11:25:30 *(beta)*
rev: 6.06e/W (Aug 31 2000) The Charlesworth Group, Huddersfield, 01484 517077 CFG92

elements. There are also 25 intact and degraded copies of an unknown gene, which may be a membrane protein, that is flanked by transposon LTRs.

Six years ago, early in the sequencing project, there were $\sim$200 genes of known function. This has risen by around 1000 genes during the time that the sequencing project has been running, and today $\sim$1200 have a known functional assignment (i.e. have been published). This is clear evidence that the policy of free access to ongoing sequencing data has already made a significant contribution to functional work, prior to completion of the genome. A further $\sim$2200 of the predicted genes showed homology to genes of known function, and $\sim$800 are conserved but of unknown function (Val Wood, personal communication).

Across the chromosomes, gene distribution is even, but there are around ten 4-8 kb gene free regions on each chromosome, which correlate with a (G+C)/GC strand switch (GC deviation). One surprising feature is a gene that encodes a peptide synthase similar to those found in bacteria such as *Streptomyces coelicolor*, this enzyme is predicted to make a four amino acid peptide, which could be an antibiotic.

There is not much evidence of duplication in the *S. pombe* genome, there are only a few tandem duplications and the telomere regions which are duplicated, this is consistent with the prediction that *S. pombe* diverged from *S. cerevisiae* prior to the proposed genome duplication in the *S. cerevisiae* lineage (Wolfe and Shields, 1997).

The distribution of protein domains in *S. pombe* and *S. cerevisiae* is broadly similar, with some notable exceptions, *S. pombe* has more WD repeat proteins (in fact more than *C. elegans*) and around half as many binuclear cluster zinc finger proteins. *S. pombe* has less sugar transporters, which could explain its less versatile growth requirements, and less ABC transporters than *S. cerevisiae*. The analysis done at the Sanger Institute has shown that *S. pombe* has around 300 genes that are conserved with eukaryotes, or with eukaryotes and bacteria, but which are not present in the *S. cerevisiae* genome (Val Wood, personal communication), and this has been confirmed by an independent study (Aravind *et al.*, 2000). Those with eukaryotic homologues are mainly splicing factors, subunits of the 'cop complex' (the signalosome) or involved in lipid metabolism. Others would cause differences in sugar, nitrogen and carbon metabolism between *S. pombe* and *S. cerevisiae* and are conserved in other fungi. Another group have roles in chromatin structure and transcriptional and post-transcriptional silencing.

### Future Aims

Valerie Wood has been working on the analysis and annotation of the *S. pombe* genome sequence at the Sanger Institute, where the Pathogen Sequencing Unit has secured funding to develop and maintain curated organic databases (GeneDB). Initially funded for *Leishmania major*, *Try-panosoma brucei* and *S. pombe*, the generic structure will be flexible enough to be used for other organisms, facilitating cross-species comparisons. The *S. pombe* database will provide added value to the raw data, incorporating all of the information from the annotation. The controlled vocabularies of GO (gene ontology) consortium (**http://www.geneontology.org**) will be integrated into the database schema, which will facilitate complex queries that reflect biological concepts. The database will incorporate search tools, which will give more flexibility, such as allowing a user to search for a novel motif not defined in existing databases (e.g. a putative transcription factor binding site), and enabling comparative analyses with other genomes. The database will eventually include information derived from functional genomics studies, such as microarray and proteomics experiments. The resource will also cover presentation of the genome sequence, with map and contig views including annotated features, such as genes. The sequence viewer will allow users to zoom in to various levels, from full chromosome, through gene structure, down to the actual sequence. In addition to carefully maintaining the genome sequence data and synchronising with the main public databases, the group plan to provide curated protein reports that will form an integral part of the database. A prototype of the protein reports is available at **http://www.genedb.org** and the *S. pombe* data can be downloaded from **http://www.sanger.ac.uk/Projects/S_pombe/ftp.shtml**. These protein reports will also allow the user to view the results of database searches (against other genomes, EST, protein and domain resources) and predictive software (for transmembrane regions, signal peptides, secondary structures etc.) displayed against the sequence. She feels that the two complete yeast genomes (*S. pombe*

Copyright © 2002 John Wiley & Sons, Ltd.

Comparative and Functional Genomics (CY74990_8.3d) 12/4/02 11:25:30 (beta)
rev: 6.06e/W (Aug 31 2000) The Charlesworth Group, Huddersfield, 01484 517077 CFG92

and *S. cerevisiae*) together will be a much more valuable resource than either could be alone. There is a large orthologous/paralogous gene set between the two (approaching 80% if we include those genes with lower homologies, but that have other supporting evidence) and cross-referenced information will be included in the database. Although a number of eukaryotic genomes are coming on line, she feels that the sheer size and more complex gene organisation of these organisms means that it may be some time before we can say that we have identified all the genes and that the predictions are highly accurate, which is a requirement for accurate interspecies comparisons. Without complete genomes, it is not possible to draw conclusions based upon gene absence, and searches for distant homologues are made more difficult. *S. pombe* will provide a complete genome, with no gaps (except in the notoriously difficult centromere and telomere regions), and highly accurate gene predictions that have been subjected to multiple rounds of manual inspection and many consistency checks. The high quality of the annotation is a testament to the contributions of the *S. pombe* community, and their continued involvement will be crucial for the resource to achieve its full potential.

A Gene naming committee has been initiated to coordinate fission yeast gene naming. The committee aims to resolve existing gene name conflicts, ensure consistent gene naming, and circulate implemented proposals to the community and public databases. The GNC can be contacted by mailing GNC@sanger.ac.uk and proposals can be viewed at **http://www.genedb.org/pombe/gene_naming_registry/**

Jürg Bähler is leading a new ICRF-funded group at the Sanger Institute that is focussing on post-genomics research with *S. pombe*. They have built a microarray that contains probes for all the genes of the fission yeast genome. 200–500 bp of each gene were amplified using gene-specific primer pairs. Computer scripts have been designed to select primers and amplicons without intron sequences and without significant homology to other regions in the genome. The array also includes 'pseudogenes', some RNA-encoding genes, mitochondrial genes, several introns, and bacterial control genes. The latest array contains ~6000 elements that are printed in duplicate onto glass slides. Numerous optimisations have been carried out to establish protocols for RNA preparation, target labelling, and hybridisation that are simple and yield reproducible signals with high sensitivity.

The group has now started biological experiments to study global gene expression profiles during the vegetative cell cycle, meiotic differentiation, and in response to oxidative stress. Genes with known regulation of expression behave as expected on the microarrays, giving confidence in the reliability of the data generated. These projects will give insights into gene function, regulatory circuits, and sequence motifs for transcriptional control both during normal cell life and in response to unfavourable conditions.

To complement and expand these studies, the group plans to build an array containing the entire sequence information of at least one chromosome (as ~1 kb long non-overlapping PCR products). These genome 'tiling' arrays will then be used to map protein-binding sites globally along whole chromosomes by probing them with DNA that co-precipitates with a DNA-binding protein of interest (ChIP technology). This will provide an alternative approach to determine the targets of transcription factors within the genome, and to investigate the dynamic behaviour of these factors through the cell cycle and other life stages. These arrays should also be useful to study other processes involving chromatin and for transcript length mapping and identifying additional ORFs.

The group is currently not in a position to provide a service for the community, but they help with access to this technology by hosting visitors from other groups, who are using their infrastructure and protocols to perform array experiments and data analysis. The first of these joint projects have now been initiated. This set up will also help to pool and compare several large datasets from various experiments that have all been performed under standardised conditions. This will allow them to take full advantage of the potential of microarray data, providing much additional information and insights, and hypotheses to follow up. These projects will create a basic dataset for future functional genomics approaches in fission yeast and will provide a starting point to characterise unknown genes and signalling pathways in detail using more traditional approaches.

Ramsay McFarlane, with Henk Braig and colleagues at the University of Wales Bangor, is establishing standard 2D techniques to study the *S. pombe* proteome. The completion of the *S. pombe* genome project and the ensuing microarray studies are providing a unique opportunity to develop a comprehensive analysis of the *S. pombe* proteome.

Copyright © 2002 John Wiley & Sons, Ltd.

Comparative and Functional Genomics (CY74990_8.3d) 12/4/02 11:25:31 *(beta)*
rev: 6.06e/W (Aug 31 2000) The Charlesworth Group, Huddersfield, 01484 517077 CFG92

This will maximise our understanding of how whole module functions are interwoven within the living cell and consequently change the context in which we study the biology of individual genes. Proteomics, and the associated technologies, are still in their infancy and the limitations of 2D gel techniques mean that it remains difficult to carry out detailed proteomic studies on organisms with large proteomes. The relatively small proteome of *S. pombe* offers a simple eukaryote model to which we can realistically apply current proteomics technology and develop the tools to make meaningful comparative studies a reality.

The *S. pombe* proteome project is now in the formative stages. Work to date has employed broad pH range isoelectric focusing and gradient SDS polyacrylamide gels. From this work, in the region of 1100 individual protein spots have been reproducibly resolved using relatively low amounts of protein on individual gels. Overlapping 'Zoom' gel systems are being employed, down to single pH range, in combination with high protein loading to determine the maximum number of spots that can be resolved for the *S. pombe* proteome using current 2D gel techniques (based on predictions from work in other microbes, we anticipate that we would have the potential to resolve in the region of 10000 spots). One of the main aims of this work is to develop techniques for *S. pombe* proteomic studies that will maximise the identification of low abundance proteins. This approach will negate the need to carry out problematic *in vivo* labelling of proteins (that may dramatically alter the biology of a cell) or complex fractionation and separation techniques (that make comparative quantification questionable at best).

Many researchers wish to apply proteomic approaches to comparative studies of different populations of cells. One of the major stumbling blocks for more wide spread use of this approach has been the poor nature of gel-to-gel reproducibility, making it difficult and time consuming to identify true differences between samples. The group at Bangor are employing a relatively new technique developed in John Minden's laboratory at Carnegie Mellon University known as difference gel electrophoresis (DIGE; Unlu *et al.*, 1997; Tonge *et al.*, 2001). This technology involves covalently labelling each protein preparation with structurally similar fluorophores possessing different excitation wavelengths. The differentially labelled samples can then be run on the same gel, and with the appropriate gel analysis instrumentation and software, one can identify true changes in proteome patterns in the absence of gel-to-gel variations. The Bangor group have synthesised their own fluorescent dyes and are currently attempting to develop new fluorescent dyes to permit multiple differentially labelled samples to be compared on a single gel. They are now using this technology to address important biological questions. During the course of the study the group aims to collaborate closely with the Sanger Institute to ensure that maximum benefit is gained from analysis of the proteome. A long-term goal is to make all the data available to the broader community through the *S. pombe* web pages at the Sanger Institute.

Susan Forsburg is an Associate Professor at the Salk Institute, her group study the control of DNA replication and the regulation of the cell cycle in *S. pombe*. She thinks that the first challenge now that the complete genome sequence has been released is to maintain and curate the data, in terms of keeping it up to date and making sure that it reflects the current state of *S. pombe* research. In her view, the Sanger Institute has done an excellent job of making the data readily available in raw form, but she feels that they need to make sure that the analytical tools, that they are currently building for the next level analysis, are easily and intuitively accessible. This is a tremendous opportunity to do detailed genome-level comparisons between *S. pombe* and *S. cerevisiae* and she would like to see the Sanger Institute and Stanford talking about an ongoing project to facilitate this analysis, which she feels will lend real insights into the subtle differences of gene behavior and evolution.

Another idea she would like to see considered is the establishment of a *S. pombe* genomics center that would work collaboratively with the community to facilitate access to genome-level tools and computing. One reason for having such a center would be that taking advantage of microarray chip technology, and having the computational skills to analyze the data produced might not be feasible for individual laboratories, but could be more practically done by a dedicated genomics center. Individual labs could collaborate with the center, and pay a user fee. She suggests that there could be a policy to publish and make the data public several months after the experiments were done.

The *S. pombe* community is relatively small, compared to the *S. cerevisiae* community, and is

Copyright © 2002 John Wiley & Sons, Ltd.

Comparative and Functional Genomics (CY74990_8.3d) 12/4/02 11:25:31 *(beta)*
rev: 6.06e/W (Aug 31 2000) The Charlesworth Group, Huddersfield, 01484 517077 CFG92

often in isolated labs, she thinks that the community have to think creatively about how to use the genome to unite *S. pombe* researchers. It is to their benefit to develop *S. pombe* as an experimental system and to give everyone access to the tools. For example, she likes the dispersed nature of the current Internet tools for *S. pombe* and feels that too much centralization can exclude people. She found that the First International Fission Yeast Meeting (held in Edinburgh in September 1999) gave her a chance to see just how many people there are working on *S. pombe* and hopes the community continues to expand, with the results of the genome project giving everyone a stake.

Iain Hagan is a Reader in the School of Biological Sciences at Manchester University and a Professor, and Group Head, at the Paterson Institute for Cancer Research. His group studies chromosome segregation at the molecular level. This highly intricate process is orchestrated by an elaborate structure called the mitotic spindle. The interest of the group centres on the organisation and function of the spindle. He feels that approaches that directly interrogate the genome sequence such as arrays and mass spectroscopy are clearly going to be of great interest to those that are interested in particular aspects of transcription and proteomics. However, he sees that the completion of the genome sequence will also have a huge impact on the traditional strengths of the organism. The strong genetics of *S. pombe* has long been exploited to take a functional approach through the use of genetic screens which identify mutants that are defective in a process of interest. Below he describes how it is now entirely possible for a postdoc or a graduate student to take on a screen that would have kept an entire lab busy for 3 or 4 years, before the sequence was determined.

The rate-limiting step in such screens has been the identification of the genes mutated in the multiple complementation groups that emerge as the screen shrinks from numerous candidate mutants to a limited number of distinct loci. Now, if the screen permits the cloning of genes by complementation of a conditional phenotype, it is trivial to clone the gene and multi-copy suppressors in a large-scale transformation. One then only needs to know the sequence of 50 bp at either end of the insert in the rescuing plasmid to find out where the sequence is in the genome. This identifies genetic markers that are physically linked to the sequence. If a mutant in such markers is readily available it can be crossed to the original mutant and so allow one to determine whether the plasmid contains the gene or a suppressor. If there are multiple ORFs on the insert of the rescuing plasmid it is trivial to use the transposon approach, such as that described by Morgan *et al.* (1996) to identify the gene of interest. The principle behind this step is simple. Bacteria containing the plasmid of interest are mated to a master strain. Transposons are mobilised and only colonies containing plasmids that have random insertions of transposons in the gene of interest grow up the next morning. A library of these clones is transformed back into the original *S. pombe* mutant strain under permissive conditions. When colonies have grown up a replica of the plate is made and incubated under restrictive conditions. If the transposon has inserted into the gene that suppresses the mutation then the colonies will fail to grow under the restrictive conditions. Plasmids are therefore re-isolated from these non-complementing colonies and sequenced to determine which ORF is disrupted using primers that read off from either end of the transposon. With this approach it is entirely feasible for a single worker to screen through several mutants and then prioritise the loci for investigation before embarking on a detailed study of the gene. In more labour intensive traditional approaches so much energy was invested in the cloning and sequencing of genes that time constraints and the pressure to publish forced workers to stay with the first one or two genes that they isolated, regardless of their relative importance to the question addressed by the screen. Now we can adopt a much wider and structured approach to genetics in *S. pombe*. The age of genomics also heralds an acceleration of the pace of classical genetics.

Dr Paul Nurse FRS is Director General of the Imperial Cancer Research Fund and was recently awarded a Nobel Prize for his work on the cell cycle. Below he talks about the implications of the sequence, both for *S. pombe* lovers and the broader genomics community.

Having the complete genome sequence will facilitate the use of tagged insertion mutagenesis strategies to isolate mutants of the 80% or so of *S. pombe* genes that have non-lethal phenotypes. Having a second uni-cellular Eukaryote genome will help us identify genes which are important for all Eukaryote cells: we have identified some of the genes which are highly conserved in all the sequenced Eukaryotes, but are absent from all the

Copyright © 2002 John Wiley & Sons, Ltd. 

Comparative and Functional Genomics (CY74990_8.3d) 12/4/02 11:25:31 (beta)
rev: 6.06e/W (Aug 31 2000) The Charlesworth Group, Huddersfield, 01484 517077 CFG92

sequenced Prokaryotes. Our approach has picked out genes involved in known Eukaryote specific systems or in pathways that differ significantly in Prokaryotes, such as compartmentation, the cytoskeleton, cell cycle control, spliceosomal proteins and certain ribosomal proteins. We will also be able to use the *S. pombe* genome to help identify those genes that are important only for multicellular Eukaryotes. Having two yeast and three animal genomes should help us look at the transition to multicellularity. *S. pombe* has many more introns than *S. cerevisiae*, with 43% of genes having at least one intron. We hope then that *S. pombe* may make a contribution to answering the question of the evolutionary origins of introns. *S. pombe* genes also have longer upstream regions on average than those of *S. cerevisiae*, which may mean that they are more complex, possibly more like those of higher Eukaryotes. We have made a big effort to unravel the sequence of the *S. pombe* centromeres. These are much larger (~300–1000-fold) and more complex than those of *S. cerevisiae*. This will offer the first opportunity to make a structural and functional analysis of larger Eukaryotic centromeres. *S. pombe* has homologues of 172 of 289 known human disease genes, and 50 of these have highly significant homology scores. Of these 50 genes, around half are involved in cancer and are also conserved in *S. cerevisiae*. Both *S. pombe* and *S. cerevisiae* are good models for the study of genome stability and the Eukaryote cell cycle, both of which are implicated in many cancers. Several of the other genes were for metabolic disorders, another area in which these yeasts should make good models.

### Web-based resources

### General Information

WWW information on *Schizosaccharomyces pombe*
**http://www.bio.uva.nl/pombe/**
This site contains a wealth of information, including a description and illustration of the *S. pombe* life cycle, an animation of *S. pombe* cell cycle and an extensive categorised list of links.

Fission Yeast Handbook (produced by the Nurse lab)
**http://www.bio.uva.nl/pombe/handbook/**
An online handbook of techniques for working with *S. pombe*, with sections on Growth, Maintenance, and Classical Genetics, Molecular Genetics, Physiology, Microscopy and Molecular Biology.

The Forsburg lab page
**http://pingu.salk.edu/~forsburg/lab.html**
This site contains an array of practical information on *S. pombe*, including an index of *S. pombe* vectors and a collection of protocols, including ones for particularly problematic areas of *S. pombe* research. A list of technical references is also available along with an impressive collection of *S. pombe* and more general yeast links.

NIH Resources for the *Schizosaccharomyces pombe* Community
**http://www.ncbi.nlm.nih.gov/Yeast/fission.html**
This site has a list of *S. pombe* links and provides access to (the now unsupported) XREFdb, a comparative database which cross references model organism genes with mammalian phenotypes.

Overview of the *S. pombe* life cycle
**http://www.teaching-biomed.man.ac.uk/ramsay/pomov.htm**
The Hagan Group at the University of Manchester use *S. pombe* to study cell cycle, here they present a guide to the process, which includes video footage. There are also links to other groups at the university and elsewhere who use *S. pombe* to study cell cycle.

*S. pombe* group at University of Copenhagen
**http://biobase.dk/%7Eonigen/pombehome.html**
This page has details of the group's work on *S. pombe* sexual differentiation and an extensive list of *S. pombe* lab websites (although it seems that several are out of date).

*Schizosaccharomyces pombe* & Yeast Link Site
**http://www.yk.rim.or.jp/~aisoai/spom.html**
This page provides a list of useful *S. pombe* and yeast pages.

### Sequencing and Databases

Sanger Institute *S. pombe* Genome Sequencing Project
**http://www.sanger.ac.uk/Projects/S_pombe/**
The *S. pombe* annotated genome sequence is available in EMBL and Genbank format from the ftp site **http://www.sanger.ac.uk/Projects/S_pombe/ftp.shtml**, see also **http://www.genedb.org**. The site

Copyright © 2002 John Wiley & Sons, Ltd.

Comparative and Functional Genomics (CY74990_8.3d) 12/4/02 11:25:32 *(beta)*
rev: 6.06e/W (Aug 31 2000) The Charlesworth Group, Huddersfield, 01484 517077
CFG92

has a BLAST server for *S. pombe* sequence searches, gene name and protein motif searches and catalogues of protein domains and functional categories. There is an ftp site for downloading DNA data and predicted protein translations and clones can be requested from the site.

*Pombe*PD – Proteome.Inc
**http://www.proteome.com/databases/index.html**
This resource holds data on 4842 proteins, 1767 of which are of unknown function. The data includes a large amount of functional information transferred from *S. cerevisiae* and *C. elegans*. The data can be searched using a gene name or keyword, or with an amino acid sequence. Using the full search form, users can retrieve categories of proteins, such as all those with ATP binding cassettes, or all those expressed in the endoplasmic reticulum, or all those with an abnormal sporulation mutant phenotype.

Cold Spring Harbor Lab *Schizosaccharomyces pombe* sequencing project
**http://nucleus.cshl.org/pombeweb/**
This page provides access to the *S. pombe* genomic sequence data generated at the Cold Spring Harbor Labs.

*S. pombe* entries in SWISS-PROT
**http://www.expasy.ch/cgi-bin/lists?pombe.txt**
This page contains a regularly updated list of *S. pombe* proteins in SWISS-PROT.

*S. pombe* Mitochondrial Genome
**http://megasun.bch.umontreal.ca/People/lang/species/spo/spombe.html**
This page from the Fungal Mitochondrial Genome Project contains a map and the sequence of the *S. pombe* mitochondrion.

### Stock Centres

The National Collection of Yeast Cultures
**http://www.ifrn.bbsrc.ac.uk/ncyc/Default.html**
Located at the Institute of Food Research in Norwich, UK, the NCYC has a wide-ranging collection of *S. pombe* strains (**http://www.ifrn.bbsrc.ac.uk/ncyc/PombeNotes.html**)

American Type Culture Collection
**http://www.atcc.org/**
The ATCC has a small collection of *S. pombe* strains and plasmids.

### Functional Genomics Projects

Fission Yeast Functional Genomics (Sanger Institute)
**http://www.sanger.ac.uk/PostGenomics/S_pombe/**
This group has been funded by the ICRF to work on various functional genomics projects, the first of which is to set up microarrays for *S. pombe*. These are glass slides, spotted with 200–500 bp PCR products for each ORF, amplified using gene specific primer pairs. The project is still in the early stages, so far, the group has made their PCR protocols available on the site and they will be putting up public data in the future.

Cell Magic World
**http://www-karc.crl.go.jp/bio/CellMagic/**
The Structural Biology Section of the Kansai Advanced Research Center have performed a large scale analysis of *S. pombe* protein subcellular localization. This page provides access to the images obtained from GFP-fusion lines. The data is presented as a list, categorized by subcellular location, or can be searched using keywords.

BLAST search against an *S. pombe* cDNA library
**http://133.63.36.123/~dclust/cgi-bin/SearchQuery.cgi**
This page provided by Dynacom (Inc.) offers a nucleotide or protein BLAST search against a library of *S. pombe* cDNAs, or entries in GenBank, SWISSPROT and other databases.

### Comparative Genomics

Genolevures Project
**http://cbi.labri.u-bordeaux.fr/Genolevures/Genolevures.php3**
This Genoscope project was a survey of the genomes of 13 yeast species representing various branches of Hemiascomycetes. Single pass sequencing of both ends of random genomic clones was used to provide a sample of each genome. Although *S. pombe* was not included in the analysis, this data will be of interest for further comparative studies with *S. pombe*.

Fission Yeast Gene Conversion Table
**http://pingu.salk.edu/~forsburg/genetable.html**
This table is a list of *S. pombe* genes and the

Copyright © 2002 John Wiley & Sons, Ltd.

Comparative and Functional Genomics (CY74990_8.3d) 12/4/02 11:25:32 (beta)
rev: 6.06e/W (Aug 31 2000) The Charlesworth Group, Huddersfield, 01484 517077 CFG92

names of their orthologues in *Saccharomyces cerevisiae*.

### *S. pombe* books

Nasim A, Young P, Johnson BF (Eds). 1989. Molecular Biology of the Fission Yeast. Academic Press.

Fantes P, Beggs J. (Eds). 2000. The yeast Nucleus. Oxford University Press.

Giga-Hama Y, Kumagai H (Eds). 1997. Foreign Gene Expression in Fission Yeast *Schizosaccharomyces Pombe*. Springer-Verlag Berlin and Heidelberg GmbH & Co. KG.

### General Yeast Books

Barnett JA, Payne RW, Yarrow D. 2000. Yeasts: Characteristics and Identification. Cambridge University Press.

Ernst JF, Schmidt A (Eds). 2000. Dimorphism in Human Pathogenic and Apathogenic Yeasts. Karger.

Heslot H, Gaillardin C. 1992. Molecular Biology and Genetic Engineering of Yeasts. CRC Press.

Kockova-Kratochvilova A. 1990. Yeasts and Yeast-like Organisms. Wiley-VCH.

Kurtzman CP, Fell JW (Eds). 1997. The Yeasts – a Taxonomic Study. Elsevier.

Phaff HJ. 1979. The Life of Yeasts. Harvard University Press.

Walton EF, Yarranton GT (Eds). 1989. Molecular and Cell Biology of Yeasts. Kluwer Academic Publishers.

Wheals AE, Rose AH, Harrison SJ (Eds). 1995. The Yeasts Vol 6: Yeast Genetics. Academic Press.

### Acknowledgements

Source for Background: Hochstenbach F. WWW information on Schizosaccharomyces *pombe*: **http://www.bio.uva.nl/pombe/**

Source for Tools for Study: Forsburg S. The Forsburg lab page: **http://pingu.salk.edu/users/forsburg/**

### References

Aravind L, Watanabe H, Lipman DJ, Koonin EV. 2000. Lineage-specific loss and divergence of functionally linked genes in eukaryotes. *Proc Natl Acad Sci U S A* **97**: 11319–11324.

Arndt GM, Atkins D, Patrikakis M, Izant JG. 1995. Gene-regulation by antisense RNA in the fission yeast *Schizosaccharomyces pombe*. *Molecular & General Genetics* **248**: 293–300.

Atkins D, Izant JG. 1995. Expression and analysis of the green fluorescent protein gene in the fission yeast *Schizosaccharomyces pombe*. *Curr Genet* **28**: 585–588.

Bähler J, Peter M. 2000. Cell polarity in yeast. In: *Frontiers in Molecular Biology: Cell Polarity*, D. Drubin (ed.). Oxford University Press.

Bähler J, Wu JQ, Longtine MS, *et al.* 1998. Heterologous modules for efficient and versatile PCR-based gene targeting in *Schizosaccharomyces pombe*. *Yeast* **14**: 943–951.

Bezanilla M, Wilson JM, Pollard TD. 2000. Fission yeast myosin-II isoforms assemble into contractile rings at distinct times during mitosis. *Current Biology* **10**: 397–400.

Brunner D, Nurse P. 2000. New concepts in fission yeast morphogenesis. *Phil Trans R Soc Lond B.* **355**: 873–877.

Clarke ML, Patrikakis M, Atkins D. 2000. Comparative analysis of artificial antisense RNA regulation in fission yeast and human cells. *Biochemical And Biophysical Research Communications* **268**: 8–13.

Danjoh I, Fujiyama A. 1999. Ras-mediated signaling pathway regulates the expression of a low-molecular-weight heat-shock protein in fission yeast. *Gene* **236**: 347–352.

Giga-Hama Y, Kumagai H. 1999. Expression system for foreign genes using the fission yeast *Schizosaccharomyces pombe*. *Biotechnol Appl Bioc* **30**: 235–244.

Hagan IM, Petersen J. 2000. The microtubule organizing centers of *Schizosaccharomyces pombe*. *Curr Op Dev Biol* **49**: 133–159.

Haraguchi T, Ding DQ, Yamamoto A, Kaneda T, Koujin T, Hiraoka Y. 1999. Multiple-color fluorescence imaging of chromosomes and microtubules in living cells. *Cell Structure And Function* **24**: 291–298.

Krawchuk MD, Wahls WP. 1999. High-efficiency gene targeting in *Schizosaccharomyces pombe* using a modular, PCR-based approach with long tracts of flanking homology. *Yeast* **15**: 1419–1427.

Morgan BA, Conlon FL, Manzanares M, *et al.* 1996. Transposon tools for recombinant DNA manipulation: Characterization of transcriptional regulators from yeast, *Xenopus*, and mouse. *Proc Natl Acad Sci USA* **93**: 2801–2806.

Moser BA, Russell P. 2000. Cell cycle regulation in *Schizosaccharomyces pombe*. *Curr Opin Microbiol* **3**: 631–636.

Nurse P. 2000. A long twentieth century of the cell cycle and beyond. *Cell* **100**: 71–78.

Pidoux AL, Uzawa S, Perry PE, Cande WZ, Allshire RC. 2000. Live analysis of lagging chromosomes during anaphase and their effect on spindle elongation rate in fission yeast. *Journal Of Cell Science* **113**: 4177–4191.

Sawin KE. 1999. GFP fusion proteins as probes for cytology in fission yeast. *Method Cell Biol* **58**: 123+

Tonge R, Shaw J, Middleton B, *et al.* 2001. Validation and development of fluorescence two-dimensional differential gel electrophoresis proteomics technology. *Proteomics* **1**: 377–398.

Copyright © 2002 John Wiley & Sons, Ltd. 

Comparative and Functional Genomics (CY74990_8.3d) 12/4/02 11:25:33 *(beta)*
rev: 6.06e/W (Aug 31 2000) The Charlesworth Group, Huddersfield, 01484 517077 CFG92

Toone WM, Jones N. 1998. Stress-activated signalling pathways in yeast. *Genes Cells* **3**: 485–498.
Unlu M, Morgan ME, Minden JS. 1997. Difference gel electrophoresis: a single gel method for detecting changes in protein extracts. *Electrophoresis* **18**: 2071–2077.
Wolfe KH, Shields DC. 1997. Molecular evidence for an ancient duplication of the entire yeast genome. *Nature* **387**: 708–713.
Wood V, Gwilliam R, Rajandream M-A, *et al.* 2002. The genome sequence of *Schizosaccharomyces pombe*. *Nature* **415**: 871–880.
Yamamoto M, Imai Y, Watanabe Y. 1997. Mating and sporulation in *S. pombe*. In: *The molecular and cellular biology of the yeast Saccharomyces*, Pringle JR (ed.). Cold Spring Harbor Laboratory Press; New York.
Zhou BBS, Elledge SJ. 2000. The DNA damage response: putting checkpoints in perspective. *Nature* **408**: 433–439.

---

*Comparative and Functional Genomics* is a cross-organism journal, publishing studies on complex and model organisms. The 'Featured Organism Article' aims to present an overview of an organism, primarily for those working on other systems. It provides background information on the organism itself and on genomics studies currently in progress, it also gives a list of web sites containing further information and a summary of the status of the study of the genome. These sections are a personal critical analysis of the current studies of the particular organism. The 'Future Aims' section is intended to be of interest to readers who work on the chosen organism and those who study other systems, and the opinions expressed therein are those of the named contributors.

Many thanks to our Section Editor for *S. pombe*, Dr Anthony Carr, for his assistance with the article and to Dr Jürg Bähler, Valerie Wood, Dr Ramsay McFarlane, Dr Susan Forsburg, Dr Iain Hagan and Dr Paul Nurse for sharing their thoughts on the future of *S. pombe* genomics research with me.

Jürg Bähler
Fission Yeast Functional Genomics
Valerie Wood
*S. pombe* Genome Project
Pathogen Sequencing Unit
The Wellcome Trust Sanger Institute
Wellcome Trust Genome Campus
Cambridge, CB10 1SA, UK
email: val@sanger.ac.uk
**http://www.sanger.ac.uk/Projects/S_pombe**
**http://www.sanger.ac.uk/PostGenomics/S_pombe**

Dr. Ramsay McFarlane
Cell & Molecular Biology Group
School of Biological Sciences
University of Wales
Memorial Building
Deiniol Road
Bangor, Gwynedd
LL57 2UW, UK
e-mail: ramsay@sbs.bangor.ac.uk

Susan L Forsburg
Associate Professor
Molecular and Cell Biology Laboratory
The Salk Institute for Biological Studies
10010 N Torrey Pines Rd
La Jolla, California 92037
e-mail: forsburg@salk.edu
**http://pingu.salk.edu/ forsburg/**

Iain Hagan
Professor
Paterson Institute for Cancer Research
Christie Hospital
Wilmslow Road
Manchester
M20 4BX
email: ihagan@picr.man.ac.uk

Paul Nurse FRS
Director - General
Imperial Cancer Research Fund
44 Lincoln's Inn Fields
London
WC2A 3PX, UK

Copyright © 2002 John Wiley & Sons, Ltd.

Comparative and Functional Genomics (CY74990_8.3d) 12/4/02 11:25:33 *(beta)*
rev: 6.06e/W (Aug 31 2000) The Charlesworth Group, Huddersfield, 01484 517077
CFG92
